# Nonlinear Biomechanical Characteristics of Deep Deformation of Native RBC Membranes in Normal State and under Modifier Action

**DOI:** 10.1155/2018/1810585

**Published:** 2018-11-19

**Authors:** Elena Kozlova, Aleksandr Chernysh, Ekaterina Manchenko, Viktoria Sergunova, Viktor Moroz

**Affiliations:** ^1^Federal Research and Clinical Center of Intensive Care Medicine and Rehabilitology, V.A. Negovsky Research Institute of General Reanimatology, 107031, 25 Petrovka Str., Build. 2, Moscow, Russia; ^2^Sechenov First Moscow State Medical University (Sechenov University), 119991, 2-4 Bolshaya Pirogovskaya st, Moscow, Russia

## Abstract

The ability of membranes of native human red blood cells (RBCs) to bend into the cell to a depth comparable in size with physiological deformations was evaluated. For this, the methods of atomic force microscopy and atomic force spectroscopy were used. Nonlinear patterns of deep deformation (up to 600 nm) of RBC membranes were studied in normal state and under the action of modifiers: fixator (glutaraldehyde), natural oxidant (hemin), and exogenous intoxicator (zinc ions), *in vitro*. The experimental dependences of membrane bending for control RBC (normal) were approximated by the Hertz model to a depth up to 600 nm. The glutaraldehyde fixator and modifiers increased the absolute value of Young's modulus of membranes and changed the experimental dependences of probe indentation into the cells. Up to some depth *h*_Hz_, the force curves were approximated by the Hertz model, and for deeper indentations *h* > *h*_Hz_, the degree of the polynomial function was changed, the membrane stiffness increased, and the pattern of indentation became another and did not obey the Hertz model. Quantitative characteristics of nonlinear experimental dependences were calculated for deep bending of RBC membranes by approximating them by the degree polynomial function.

## 1. Introduction

The mechanical properties and structural organization of membranes determine the functional state of red blood cells (RBCs). Deformability is one of the key physiological and biophysical indicators of RBC [1]. Changes of the mechanical characteristics of cell membranes can lead to a decrease in the rate of capillary blood flow and to development of stagnant phenomena in the microcirculation, and it can also reduce the amount of oxygen delivered to the tissues.

During circulation, RBCs are constantly deformed, passing through narrow capillaries [2, 3]. Elastic properties of RBC are largely determined by the stiffness of their membranes and the state of the cytoskeleton lining the inner side of the cell [4]. In studies of RBC membrane stiffness, Young's modulus is often determined at probe indentation to depths of 10–50 nm [5]. But, since RBCs undergo significant deformations in the capillaries, it is of particular interest to study the nonlinear laws of membrane deformation into native cells to depths comparable in size with the values of their physiological deformations (0.5 *μ*m and more).

A true estimation of the elastic properties of RBC membranes can be obtained only by measurement of native cell properties. In this case, the result will be the closest to the characteristics of a living biological object.

Measurement of the mechanical properties of membranes of native blood cells is a technically and methodically complex task. During the scanning of cells in a liquid, they can be removed by a probe. To exclude this, fixators are used: glutaraldehyde, ethanol, and osmium tetroxide [6–9]. However, fixators introduce changes in the structure of cells, which in turn can lead to a shift in the estimation of Young's modulus of membranes. The stiffness of RBC membranes is also substantially dependent on the influence of oxidants, agents of intoxication, and various diseases [3, 7, 10–15]. Estimates of the absolute values of Young's modulus of RBC membranes in works that studied this problem differ dozens of times [16–19]. Therefore, the study of the ability of native RBC membranes to be bent to large depths after the action of external factors of various natures is an important biophysical and medical problem.

In this work, the stiffness of the RBC membrane was measured under physiological conditions using atomic force microscopy (AFM), *in vitro*. The main method for quantifying the mechanical characteristics of cellular surfaces is atomic force spectroscopy (AFS). Both visualization (AFM) and measurement of cell membrane stiffness (AFS) are realized on one set of equipment and allow obtaining results with the highest resolution available in modern studies [5].

The aim is to study nonlinear mechanical characteristics of deep deformation of native RBC membranes in normal state and under the action of modifiers, *in vitro*.

## 2. Materials and Methods

### 2.1. The Scheme of Experiments

**Figure 1:**
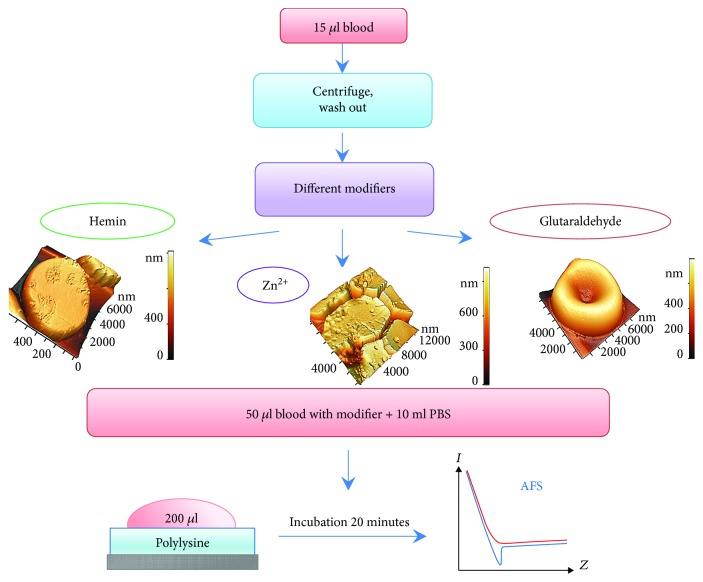
Scheme of experiments.

Experiments *in vitro* were carried out according to the scheme shown in Figure 1. In the first stage of the experiment, a suspension of erythrocytes was prepared. For this, 150 *μ*l of fresh human blood was centrifuged at 3000 rpm during 5 minutes to separate blood cells from plasma. Plasma was removed, and the volume was brought to that of the original blood sample with PBS, pH 7.4 (PBS Tablets, MP Biomedicals, USA). Hence, in suspension, RBC concentration was the same as in initial blood. The RBCs were washed three times in PBS. In the second stage of the experiment, various modifiers were added to RBCs.

### 2.2. RBC Suspensions

Blood sampling was carried out in microvettes with EDTA (Sarstedt AG and Co., Germany) during a prophylactic examination from 8 donors. All experiments were carried out in accordance with guidelines and regulations of the Federal Research and Clinical Center of Intensive Care Medicine and Rehabilitology, V.A. Negovsky Scientific Research Institute of General Reanimatology, Moscow, Russian Federation. All experimental protocols were approved by this Institute.

### 2.3. Modifiers

Studies of local stiffness of RBC membranes were carried out for the native RBCs (control) and native cells after the action of modifiers. We used such agents as glutaraldehyde (GA) (RBC membrane fixator), hemin (natural oxidant), and Zn^2+^ (heavy metal ions). Glutaraldehyde modifies complexes of actin and band 3 (crosslink of cells proteins) [8], heavy metal ions Zn^2+^ are binding to protein structures causing their aggregation [20], and hemin can destroy spectrin, influence on band 4.1, weaken connection spectrin-band 4.1, and weaken the stability of the membrane cytoskeleton [21].

In experimental series I, dry hemin (Sigma, USA) was used to prepare the work solution. First, 200 mg of NaOH was dissolved in 10 ml of distilled water and solution A was obtained. Then 50 mg of hemin powder was dissolved in 1 ml of solution A and 5 ml of distilled water was added, and solution B was obtained. 50 *μ*l (H50) or 80 *μ*l (H80) of solution B were added into microvettes with blood. The incubation time was 60 minutes.

In experimental series II, 180 mg of ZnSO_4_ (Sigma, USA) was dissolved in 10 ml of phosphate buffer PBS, pH 7.4, to prepare the modifier. Then, 10 *μ*l of the resulting solution was added to 100 *μ*l of RBCs, which was previously centrifuged at 500 rpm for 5 minutes to remove plasma. The concentration of Zn^2+^ ions in blood *in vitro* was 4 mM.

In experimental series III, 0.2% and 1% glutaraldehyde (Panreac Quimica SLU, Spain) was used as a modifier. A solution of glutaraldehyde was added into blood in a volume ratio of 1 : 1. These suspensions were marked correspondingly as GA 0.2% and GA1%. The cell suspension was incubated up to 4 minutes.

### 2.4. Preparation of RBC Samples for AFS

Сover glasses were used as the substrate for formation of RBC monolayers. The glasses were previously left in solution of polylysine at a concentration of 1 mg/ml in a Petri dish for 12 hours, then the glasses were air-dried for 2 hours. Polylysine creates positive charges over the substrate that interacts with the negative charges found over the RBC membrane [22].

The method of sedimentation was used to prepare the monolayer. To do this, 50 *μ*l of RBC was diluted in 10 ml of phosphate buffer PBS. Then, 200 *μ*l of erythrocyte suspension was dropped on glass with polylysine and left for 20 min for sedimentation of the cells, the drop does not dry out. The resulting sample was washed in PBS during 10 seconds and scanned by AFM. If native RBC is displaced from the substrate, the optical method of cell indication was used.

### 2.5. Atomic Force Microscopy

The atomic force microscope (AFM) NTEGRA Prima (NT-MDT, Russian Federation) was used to obtain сell and membrane images and to measure local stiffness of RBC membranes in a liquid. To acquire images, NSG01 cantilevers (Nanosensors, Switzerland) with a force constant of 5 N/m and tip radius of 10 nm were used. The numbers of scan points were 512 and 1024. Measurements of local stiffness of RBC membranes were performed by ASF on the vertical displacement of a piezoscanner, where the sample was placed [14, 23]. To measure the deformation of the membrane, the type SD-R150-T3L450B-10 (Nanosensors, Switzerland) cantilever was used. The radius of the cantilever probe was 150 nm, the coefficient of elasticity was 1 N/m, the probe height was 15 *μ*m, and the resonance frequency was 21 kHz.

To measure stiffness, RBCs were scanned in an AFM field 100 × 100 *μ*m^2^; a group of cells was selected for the study and scanned in the field of 30 × 30 *μ*m^2^. Then, in the atomic force spectroscopy mode, a marker was placed on the cell image and the region was exposed to an indenter (probe) with the force *F*. The characteristics and peculiarities of deep penetrations of the probe into the membrane were studied. That is, curves of the piezoscanner's approach was used in the analysis of the experimental data, *I*(*z*) and correspondingly *F*(*h*).

### 2.6. The Process of Measurements and Probes

*The elasticity coefficient K* of the cantilever should be comparable with the mean stiffness coefficient *K*_m_ of the cell membrane [24, 25]. If *K*_m_ ≫ *K* (glass), then a dependence between the force *F* and the deviation of the cantilever *L* is determined by Hooke's law, *F* = −*KL*, and the force curve is linear. In this case, *L* = *Z* and indentation depth *h*⟶0. If the probe interacts with the membrane and *K*_m_ ≪ *K*, then *h*⟶*Z*, and the membrane does not exhibit elastic properties. If the probe interacts with membrane and *K*_m_ ≈ *K*, then *F* = −[1/*K* + 1/*K*_m_ (*h*)]^−1^ *L*, and the probe bends the cell membrane to a depth of *h* (*h* < *Z*).

Based on the experimental data, the optimal value of *K* for measuring the local stiffness of RBC membranes is in the range of 10–50 N/m for dry cells and 0.05–1.8 N/m for native cells.

*The time of probe indentation* was at least 5–10 seconds in our work. Fast indentation can change the mechanical reaction of RBC membranes [19]. The aim of the study was not to investigate the change in force curves as a function of the time of penetration. Therefore, long penetration times of the indenter were chosen. The speed of indenter penetration was 0.05–1 *μ*m/s. With such penetration rates, no effects of viscosity-related friction forces were observed.

*The tip radius R* should be not less than 50–80 nm. A small *R* (10–30 nm) leads to probe penetration into the membrane structure [1, 25]. As a result, rupture of the membrane is possible. For the registration of the *K*_m_ membrane, it is necessary for *R* to be larger than the size of the spectrin matrix element.

*The tip height* should be not less than the height of the RBC (2–10 *μ*m).

*Cantilever calibration* on highly rigid material (e.g., glass) must be carried out before and after each series of measurements [14, 23]. The empirical force curve is the dependence of photodiode deflection current *I* on the magnitude of the vertical displacement *Z* of the piezoscanner, *I*(*Z*). These parameters can be varied during measurements on AFM, depending on the purpose and object of the study. For our experiment, the maximum limits of piezoscanner shift were set as *Z*_max_ = 2000 nm and current *I*_max_ = 0.3–0.6 nA.

### 2.7. Statistical Analysis

In the work, the following groups of RBC from donors were analyzed: control—8 donors, after exposure by GA0.2%—3 donors, GA1%—3 donors, H50—3 donors, H80—3 donors, and Zn^2+^—2 donors. In total, 22 RBC samples were analyzed. For each sample, 50 native cells were analyzed. For them, experimental force curves were registered. Moreover, experimental dependences *F*(*h*) were created. In total, the quantitative characteristics of 1100 native cells were analyzed and calculated. All standard statistical calculations of all obtained experimental results and mathematical modeling were performed by Origin 9 (OriginLab, Northampton, MA). One-way ANOVA was used to determine statistical significance.

## 3. Results

### 3.1. Mechanical Characteristics of RBC Membrane Bending

Elastic properties of RBC membranes, namely, the ability of the membrane to bend into the cell under the action of the applied force, was estimated from empirical force curves. These characteristics were obtained by AFS. The local stiffness of RBC membranes was estimated by Young's modulus *E* (N/m^2^), calculated from the Hertz model [26]

(1) $$\begin{array}{c} F=\frac{4}{3}ER^{0.5}h^{1.5}, \end{array}$$

where *E* is Young's modulus of the material, *R* is the tip radius, and *h* is the deformation depth (bending) of the membrane. Such estimation is used for blood cell membranes [9, 25, 27–30].

**Figure 2:**
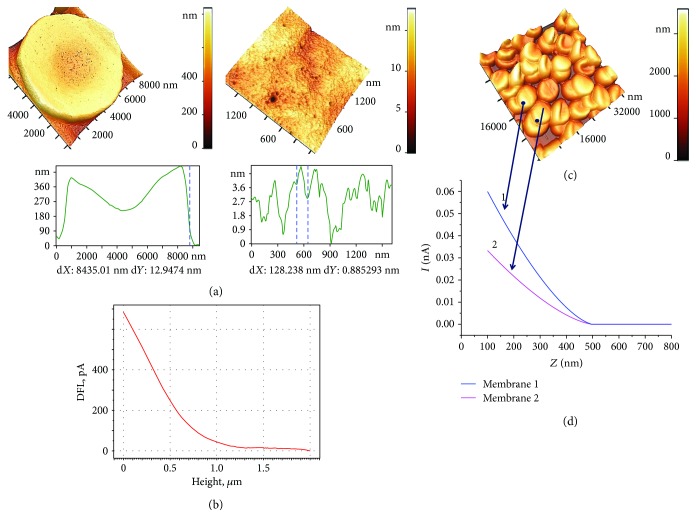
AFM images of RBC and force characteristics of the membranes. (a) 3D image of dry cells, 10 × 10 *μ*m^2^, of membrane fragments, and corresponding surface profiles. (b) Directly measured data *I*(*Z*). (c) 3D images of native cells in a liquid medium, 50 × 50 *μ*m^2^. Arrows—points of application of force *F* from the probe. (d) Force curves *I*(*Z*) after smoothing. I: the photodiode current (nA); Z: vertical displacement of the piezoscanner (nm).

**Figure 3:**
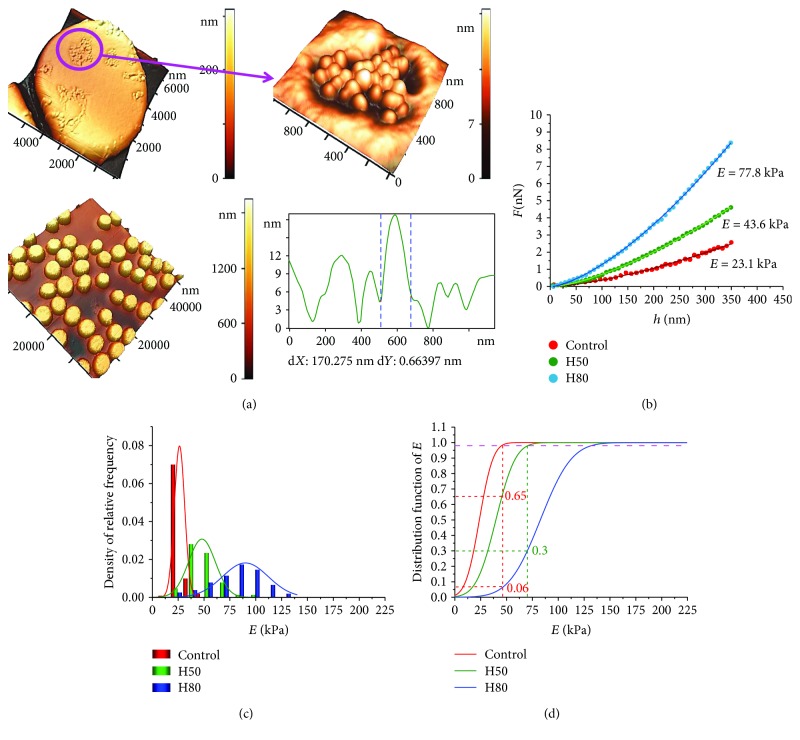
The influence of hemin on RBC membrane stiffness. (a) AFM images of RBCs, 3D image cells, 50 × 50 *μ*m^2^, of a single cell, 10 × 10 *μ*m^2^, of a membrane defect in the form of a grain domain, and its profile. (b) Experimental curves *F*(*h*) for one RBC control, for hemin H50, and for H80, and their approximation by the Hertz model. (c) Histograms of density of relative frequency of Young's modulus *E* for control, H50, and H80, approximated by the normal law of the Gaussian distribution. (d) Distribution function of Young's modulus *E* (approximation by the normal Gaussian law) for the control and after the influence of H50 and H80.

**Figure 4:**
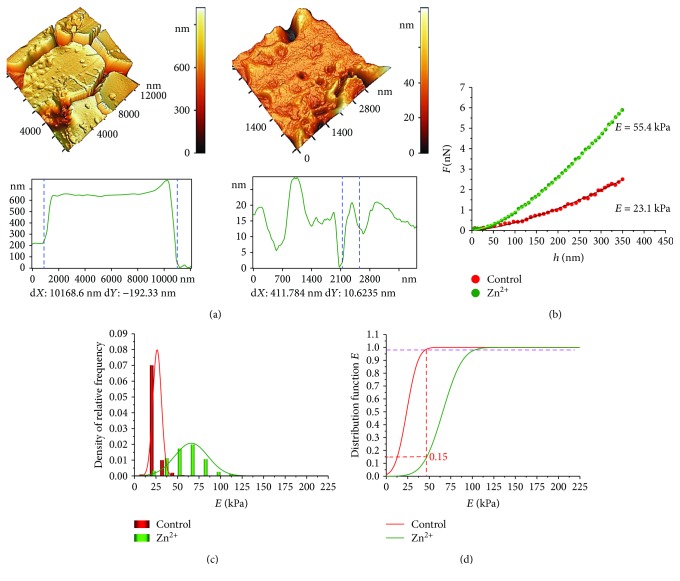
Influence of Zn^2+^ ions on RBC membrane stiffness. (a) AFM 3D images of RBC, 10 × 10 *μ*m^2^, of a membrane topological defect, 3 × 3 *μ*m^2^, and their profiles correspondingly. (b) Experimental curves *F*(*h*) for membranes after control and zinc ion action. (c) Histograms of the density of relative frequency of Young's modulus *E* for the control and zinc ion action, approximated by the normal law of Gaussian distribution. (d) Distribution function of Young's modulus *E* (approximation by the normal Gaussian law) for the control and after influence of Zn^2+^.

AFS can be performed both on dry cells and on native RBC. Dry cells are easily fixed on a substrate (glass, mica, etc.), and, most importantly, they can be scanned with a thin (~10 nm) cantilever probe for analyzing their membrane nanostructure (Figures 2(a), 3(a), and 4(a)). In these cases, the resolution limit is a part of nanometers.

The method of sample preparation of native blood cells is described in Methods. It is almost impossible to record the nanostructure of native cells because of insufficient resolution of images. This is determined by the passage of the AFM laser beam through the liquid medium in which the RBCs are located. Another reason for the loss of high resolution is the use of probes with a large radius. In our works, the tip radius was 150 nm. Therefore, the image of native cells was blurred, but enough to set the points of tip probe action (Figure 2(c)).

When the piezoscanner rises, the cantilever probe acts on the application point of the RBC membrane with a given force *F*. As a result, the laser beam deflects, forming a photodiode electrical current *I*. In Figure 2(b), the primary (directly measured) data *I*(*Z*) are shown.

In Figure 2(c), 3D images of native cells in a liquid medium, 50 × 50 *μ*m^2^, are shown, and arrows indicate point force *F* application from the probe. For two cells, force curves *I*(*Z*) after smoothing by software are represented (Figure 2(d)). The intersection of the empirical curve and the level which is high than the baseline value of0.02*∆I*were established as contact point*Z*_0_for further study. In this point, *I*′(*Z*) > 0. The value *∆I* was set as *∆I* = *I*_max_ − *I*_baseline_.

To further analyze the probe indentation process and to calculate Young's modulus *E*, it is necessary to pass from the empirical function *I*(*Z*) (Figures 2(b) and 2(d)) to *F*(*Z*) and *F* (*h*), where *F* is the force acting on the sample and *h* is the probe (tip) indentation depth, or membrane bending. The transition from the photodiode current to the interaction force was described earlier [5].

**Figure 5:**
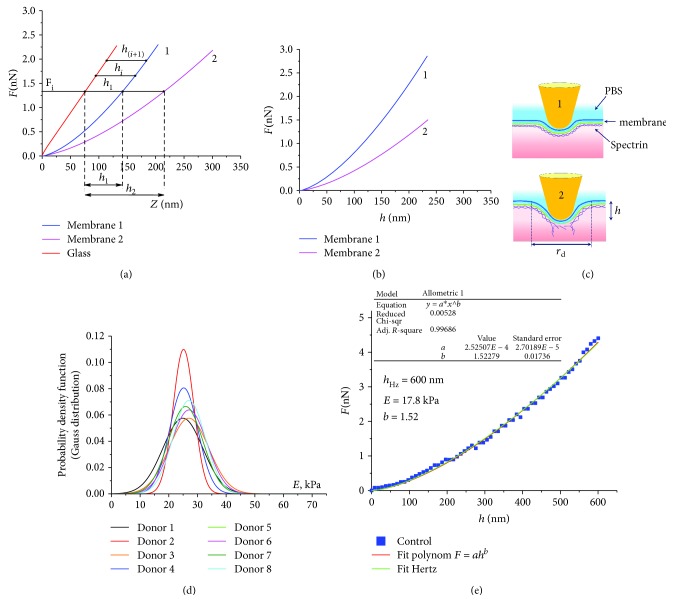
The process of RBC membrane bending under a probe action, and the construction of functions *F*(*h*). (a) Experimental curves *F*(*Z*) for highly stiff substance (glass), membranes of cells 1 and 2. (b) Experimental curves *F*(*h*) for cell membranes 1 and 2. (c) Bending of membranes under the action of force *F* for stiff (1) and soft (2) membranes; *F* is the force acting on the membrane from the probe, *Z* is the vertical displacement of the piezoscanner, *h* is the depth of the membrane bending into RBC, PBS is the phosphate buffer solution, and *r*_d_ is the bending radius of the membrane. (d) Gaussian probability density functions of RBC membrane Young's modulus *E*, for 8 healthy donors. (e) The experimental curve *F*(*h*) for one control (normal) RBC, its approximation by the Hertz model, and the polynomial *F* = *ah*^*b*^.

The functions *F* (*Z*) for glass and membranes of two different RBCs are shown in Figure 5(a). The difference between the *Z* coordinates for glass and the corresponding membrane (1 or 2) is the value of the membrane bending *h* into the cell due to probe action. From Figure 5(a), it follows that membrane 2 is softer than membrane 1, and therefore the bending of membrane 2 at a fixed force *F* is greater than the bending of membrane 1: *h*_2_ > *h*_1_ (Figure 5(c)). The force *F* determines the deviation *L* of the cantilever and simultaneously the deformation of the membrane itself, that is, penetration of the probe into cell to a depth *h* (Figure 5(a)):

(2) $$\begin{array}{c} h=Z–L. \end{array}$$

As the probe bends the membrane into RBC, the curve *F* (*Z*), for example, in Figure 5(a), can go to a straight line parallel to the glass straight line. This will mean that the process obeys Hooke's law, and the probe will stop penetrating into the cell structure, *h*⟶const and Δ*h*⟶0.

The dependence *F*(*h*) is carried out using specialized software developed by the authors.  Figure 5(b) shows an example of the functions *F* (*h*) for two membranes 1 and 2.

**Figure 6:**
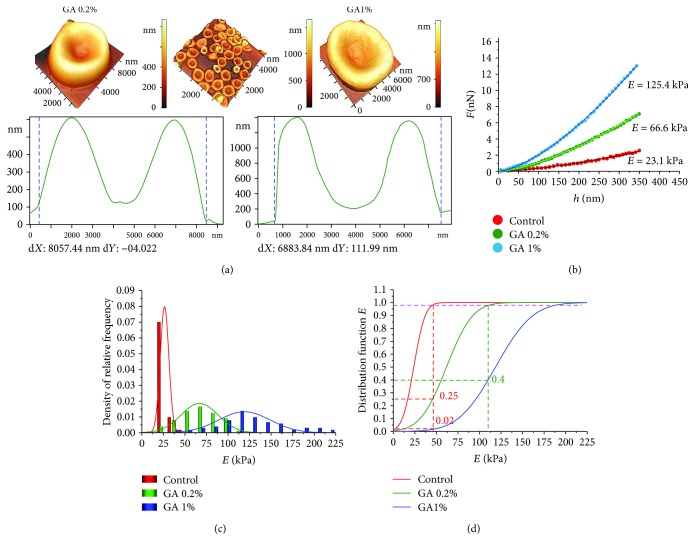
Influence of glutaraldehyde on RBC membrane stiffness. (a) AFM images of RBC, 50 × 50 *μ*m^2^ and 10 × 10 *μ*m^2^, after the action of 0.2% and 1% glutaraldehyde and their profiles, respectively. (b) Experimental curves *F*(*h*) for control cell, for cell after GA0.2%, and for GA1% action, and their approximation by the Hertz model. (c) Histograms of the relative frequency density for control RBCs and under the action of GA 0.2% and GA1% on cells, approximated by the normal law of the Gaussian distribution. (d) Distribution functions of Young's modulus *E* (approximation by the normal Gaussian law), for control and action of GA 0.2% and GA1%, correspondingly.

RBCs of each donor (in normal state and under different effects on blood) had initially different absolute values of the membrane modulus *E*. Therefore, an adequate estimation of RBC membrane stiffness of a given donor required the formation of a cell ensemble and further statistical processing. For each donor, 50 functions *F*(*h*) were carried out by measurement of 50 cell force curves. For each function, Young's modulus was calculated according to 1; histograms of relative frequency density of the modulus *E* were plotted. The data were approximated by the Gaussian probability density function (Figures 3(c), 4(c), 5(d), and 6(c)).

### 3.2. Deformation (Bending) of Normal RBC Membranes

The results of measurements of Young's modulus *E* under bending of RBC membranes (*h* = 600 nm) for 8 healthy donors (normal, or control data) are shown in Figure 5(d) according to the sample means *E*_m_, and the standard deviations are presented in Table 1.

Approximation of the control experimental curves *F*(*h*) by the Hertz model (1) was carried out.  Figure 5(e) shows an example of one of these curves.

The same figure shows the approximation of the experimental graph by a polynomial of the form

(3) $$\begin{array}{c} F\left(h\right)=ah^{b}. \end{array}$$

The approximation curve *F*(*h*)_theor_ by the standard method of nonlinear fitting of the experimental data was used. The status of the dependent variable was assigned to the experimental data function  *F*(*h*)_exper_. The degree *b* and coefficient *a* of the polynomial (3) were unknown variables. Unknown coefficients *b* and *a* are the parameters of the model, and they are chosen by the statistical program so that the theoretical curve *F*(*h*)_theor_ describes the experimental data *F*(*h*)_exper_ in the best way (*R*^2^ > 0.95). The condition *a*_*i*_ ≥ 0 must be satisfied in the approximation. The degree *b* and the coefficient *a* of the polynomial (3) were chosen by nonlinear fitting of the experimental curves (OriginLab, Northampton, MA).

Approximation by function (3) was carried out for all curves *F*(*h*) (50 for each RBC sample). Depths *h*_Hz_, to which the curves *F*(*h*) were adequately approximated by the Hertz model (*b* = 1.50 ± 0.02), were calculated for each experimental curve. That is, the degree *b* was used as criterion for the adequacy of the Hertz model. Approximation of empirical data *F*(*h*) represented in Figure 5(e) was obtained at *h* = 600 nm: *b* = 1.52, *E*_m_ = 17.8 kPa.

All the obtained control force curves *F*(*h*) were adequately approximated by the function (1) and the polynomial (3) with the degree *b* = 1.50 ± 0.02 at the level of the determination coefficient *R*^2^ ≥ 0.95.

### 3.3. The Action of Glutaraldehyde on RBC Membranes (*h* ≤ *h*_Hz_)

Glutaraldehyde (GA) is used as a fixator for RBC membranes [7–9, 16]. Therefore, the work analyzes the effect of GA at concentrations 0.2% and 1% on Young's modulus of RBC membranes. The experimental data are shown in Figure 6.  Figure 6(a) shows the AFM images of cells after the GA action.

With increasing GA concentration, Young's modulus *E* increased, and the histogram of the relative frequency density shifted towards larger values of *E* (Figure 6(c)). Values of *E* for GA1% are statistically different from control and from GA0.2% data at *p* < 0.01.

For a detailed analysis of membrane stiffness changes under GA action in different concentrations on RBC, the distribution function *F*_d_(*E*) was constructed.

(4) $$\begin{array}{c} F_{d}\left(Е\right)=\int_{-\infty }^{Е}f\left(Е\right)dЕ. \end{array}$$

It was assumed that the histograms of relative frequency density (Figure 6(c)) are approximated by the normal Gaussian distribution law *f*(*E*). To estimate the proportion of RBC on which the modulus *E* increased, the level of 0.98 was indicated on curves of functions (4). At this level, it was determined which part of the cells retained *E* corresponding to control data. So, after GA0.2% action, 25% of cells kept the control membrane stiffness. The membrane stiffness of 75% of cells was increased. For GA1%, the percentage of cell membrane with control *E* was only 2%. At the same time, for GA1%, 40% of cells have the same values *E* as for GA0.2%.

The experimental curves *F*(*h*) (Figure 6(b)) were approximated by the Hertz model (1) and the polynomial (3) for GA0.2% and GA1%. Fifty functions *F*(*h*) for each donor and for each concentration of GA were analyzed, with a total 300. The quantitative estimations of Young's modulus are shown as an example for one donor in Figure 6(b): for control *E*_m_ = 23.1 kPa, for GA 0.2% *E*_m_ = 66.6 kPa, and for GA 1% *E*_m_ = 125.4 kPa.

### 3.4. The Action of Hemin of the RBC Membrane (*h* ≤ *h*_Hz_)

Hemin is a natural oxidant of biological structures and, in particular, an oxidizer of RBC membranes. The action of hemin H50 and H80 was studied.  Figure 3(a) shows AFM 3D images of cells (50 × 50 *μ*m^2^), of a single cell (10 × 10 *μ*m^2^) after the hemin action. Also, there are shown images of topological nanodefects in the membrane (1.2 × 1.2 *μ*m^2^) and their profiles. After hemin action on the blood, typical topological nanodefects in the form of domains with grain-like structures are formed in membranes [21]. Also, hemin may change simultaneously the elastic properties of RBC membranes.

An increase in the hemin concentration caused the growth of Young's modulus *E* (Figure 3(b)). So for H50 at the depth of *h* = 300 nm, *E* increased by 1.9 times in comparison with the control, and for H80 3.4 times.

Figure 3(c) shows histograms of density of the relative frequency of Young's modulus *E* for the control, H50, and H80, which are approximated by the normal Gaussian distribution law. The distribution of H50 and H80 is statistically different from the control and among themselves at level *p* < 0.01.

The distribution function *F*_d_ in accordance with 4 and the Hertz model (1) for control, H50, and H80 are shown in Figure 3(d). For H50, 65% of cells retained Young's modulus *E* the same as in the control. For H80, this fraction was 6%. For H80, in 30% of cells, modulus *E* was kept at the same level as after H50 action.

The experimental curves *F*(*h*) for H50 and H80 action were adequately approximated by the Hertz model (1) and the polynomial (3) with *b* = 1.50 ± 0.02, up to membrane bending to *h* = 350 nm (Figure 3(b)). Fifty functions *F*(*h*) were analyzed for each donor and for each hemin concentration, with a total of 300.

### 3.5. The Action of Zn^2+^ Ions on RBC Membranes (*h* ≤ *h*_Hz_)

Heavy metal ions, for example, zinc ions, cause membrane defects [20] and can increase their stiffness.

The AFM images of RBCs, membrane nanodefects, and their profiles after the action of Zn^2+^ (concentration 4 mM) are shown in Figure 4(a). Such concentration was chosen to obtain significant nanodefects in red blood cell membranes [20].

The functions *F*(*h*) and the histograms of the relative frequency density approximated by Gauss's law are represented in Figures 4(b) and 4(c), correspondingly. Modulus *E* for control and zinc influence samples are statistically different at level *p* < 0.01.

Figure 4(d) shows the distribution functions *F*_d_(*E*) (4). After zinc ions' action, Young's modulus was maintained at the control level for 15% of cell membranes. The rest of the RBC membranes became stiffer by 2 times and more.

### 3.6. Deep Bending of RBC Membranes (*h* ≥ *h*_Hz_)

In all studies, force *F* was chosen so that both in the control RBCs and under the action of the modifiers the probe was penetrated into the cell (membrane bending) to a depth *h* ≥ 600 nm. Up to a certain depth *h*_Hz_, the degree of the polynomial (3) was preserved at the level *b* = 1.5. After *h*_Hz_, the degree may differ from 1.5. It was denoted by *b*_*n*_, and the coefficient of the polynomial (3) is, respectively, *a*_*n*_. All parameters of the curves *F*(*h*)—*E*, *h*_Hz_, *b*, *b*_*n*_, *a*, and *a*_*n*_—were calculated to the bending depth up to *h* = 600 nm.

In the control cell membrane (Figures 5(d) and 5(e)), practically all the *F*(*h*) curves were approximated by the Hertz model from 0 to 600 nm at the level of the criterion *R*^2^ = 0.95.

**Figure 7:**
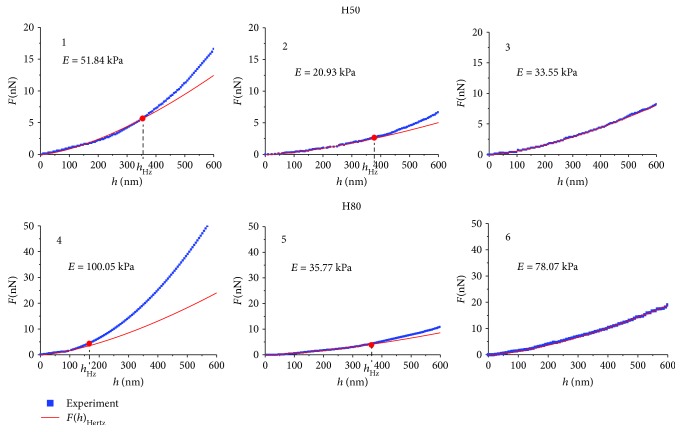
Experimental curves *F*(*h*) and functions of their approximation by the Hertz model for hemin H50 (nos. 1, 2, and 3) and hemin H80 (nos. 4, 5, and 6); *h*_Hz_ is the boundary depth of membrane bending, deeper by which approximation by the Hertz model becomes inadequate.

**Figure 8:**
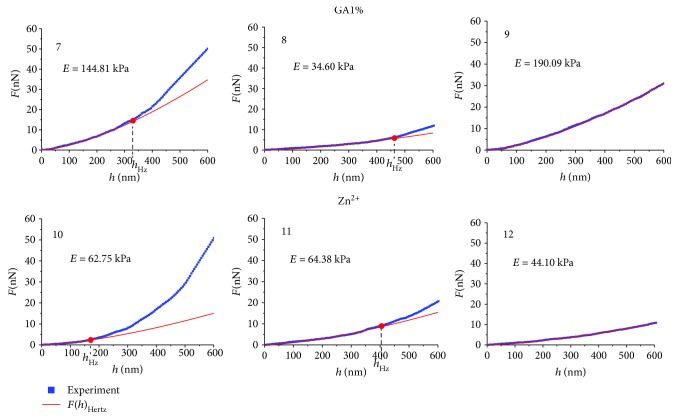
Experimental curves *F*(*h*) and functions of their approximation by the Hertz model for glutaraldehyde GA1% (nos. 7, 8, and 9) and Zn^2+^ ions (nos. 10, 11, and 12); *h*_Hz_ is the boundary depth of membrane bending, deeper by which approximation by the Hertz model becomes inadequate.

Under the modifier influence, this situation changed. On the empirical dependence of *F*(*h*) at the indentation depth *h* > *h*_Hz_, the degree *b*_*n*_ of the approximation polynomial (3) was changed. In Figures 7 and 8, three examples of empirical curves *F*(*h*) are shown for the action of hemin H50, H80, glutaraldehyde GA1%, Zn^2+^ ions on the blood, and their approximation by the polynomial (3). In Table 2, the numerical parameters of these graphs are presented.

Statistical data of the values *E* for *h* < *h*_Hz_ for control samples and for samples after action of agents are shown in Table 3. Also, there are indicated corresponding values *h*_Hz_.

In a number of cases, the degree of the polynomial (3) ranged from 1.48 to 1.52. In these cases, the entire curve *F*(*h*) obeyed the Hertz model. Such examples are given for H50 (Figure 7 and Table 2, no. 3), for H80 (Figure 7 and Table 2, no. 6), for GA1% (Figure 8 and Table 2, no. 9), and for Zn^2+^ (Figure 8 and Table 2, no. 12). The degree *b*_*n*_ of the polynomial (3) after *h*_Hz_ was less than 1.5 in 92% of cases and was in the range 1.01–1.45. In example no. 8 (Figure 8(a)) under the action of GA1%, *h*_Hz_ = 463 nm, and *b*_*n*_ = 1.03. This degree indicates that this dependence *F*(*h*) was linear after *h*_Hz_. *F*(*h*) obeyed Hooke's law. In nine cases, the degree was *b*_*n*_ > 1.5. So in example no. 10, after the Zn^2+^ action, *h*_Hz_ = 169 nm and *b*_*n*_ = 1.94, that is, almost parabolic function.

The values of the membrane bending *h*_Hz_, up to which *F*(*h*) obeyed the Hertz model, and after which point the degree of the approximating polynomial was varied, lay in a wide range. In the given examples (Figures 7 and 8), the range of *h*_Hz_ was from 169 nm to 463 nm. If *h*_Hz_ = 600 nm, this means that the total curve is approximated by the Hertz model. In all experiments, Young's modulus *E* was calculated by the Hertz model to the depth *h*_Hz_. These data are indicated in Figures 7 and 8 and in Tables 2 and 3.

With increase in the penetration depth of the probe into the sample *h* > *h*_Hz_, the modulus of elasticity was increased (Figures 7 and 8); experimental data (blue) are higher than corresponding function data (red).

To estimate the increase in modulus *E* after point *h*_Hz_, function *F*(*h*) was approximated in the interval *h*_Hz_ = 600 nm by a linear function. So for H50, after *h*_Hz_= 385 nm, Young's modulus *E* increased 1.6 times (curve 2). For H80 after *h*_Hz_ = 170 nm, modulus *E* increased by 3.3 times (curve 4). For GA1% after *h*_Hz_ = 329 nm, modulus *E* increased by 1.8 times (curve 7), and for Zn^2+^ after *h*_Hz_ = 169 nm, modulus *E* increased by 5.5 times (curve 10). These are only the approximate estimations of the increase in modulus*E*. It is not possible to calculate *E* exactly on this interval, since *b*_*n*_ has different values for each curve than the others. To create a single model for all different *b*_*n*_ is not possible.

## 4. Discussion

### 4.1. Elastic Properties of RBC Membranes

In this work, we estimated the membrane's ability to bend into the cell, by atomic force spectroscopy [23] with large radius probes (*R* = 150 nm). In all experiments, only native cells were used. Scanning and obtaining of the force curves *I*(*Z*) were carried out only in a liquid medium, namely, buffer solution. It is known that the membrane stiffness strongly depends on the method of sample preparation. So the stiffness of the dry RBC membrane can reach tens of megapascals and more [31]. Moreover, the stiffness of the native cell membrane is of the order of several tens of kilopascals, that is, three or more orders less [5, 30]. Therefore, the first task was to develop a method to obtain samples of native cells on a substrate for further scanning. It was assumed that the use of fixatives and membrane modifiers for the RBCs is unacceptable. The solution of this problem is described in the section Materials and Methods.

Membrane fixatives and modifiers, natural oxidants, and heavy metal ions significantly increased the RBC membrane stiffness.

*Glutaraldehyde as an RBC fixator* at a concentration of 0.2% increased Young's modulus by 2.9 times and at a concentration of 1.0% by 5.4 times (Figure 6(c)).

*Hemin* is a natural oxidant. The action of hemin on blood leads to an increase in Young's modulus by 3 or more times (Figure 3(a)).

Zn^2+^ ions also increased Young's modulus of RBC membranes by 2.4 times compared to control cells.

Therefore, when GA is used as an RBC fixator in studies of body pathologies (oxidative processes), of exogenous intoxication (metal ions), the obtained absolute values of Young's modulus will always be shifted upwards. Such values cannot be true and can only be used as comparative values.

### 4.2. Young's Modulus of Native RBC in Control

Control cells were cells of healthy people of both sexes from 20 to 35 years of age. All absolute values of Young's modulus (for 8 people) in our experiment were from above 11 kPa to 41 kPa. The mechanical properties of RBC membranes, in particular their Young's modulus, can be used in clinical practice as a quantitative criterion for assessing the state of blood cell membranes. If *E* lies within these limits, then we can assume that the deformability of RBC is normal. If the values of Young's modulus differ from the indicated limits, then such cells are subject to additional studies.

### 4.3. Deep Bending of RBC Membranes (*h*_Hz_ − 600 nm)

A characteristic peculiarity of dependences *F*(*h*) for deep bending of RBC membranes is that approximating polynomial degree *b*_*n*_ (3) may be changed at depths larger than *h*_Hz_, *h* > *h*_Hz_ (Figures 7 and 8, Table 2).

But in normal, more than 92% of all RBC membranes of 8 donors gave an empirical curve adequately approximated by the Hertz model even at depths up to 600 nm with a determination coefficient *R*^2^ ≥ 0.95. Additional frictional forces did not arise (Figure 5(c)).

Under influence of modifiers, the increase in Young's modulus at *h* > *h*_Hz_ was determined by additional resistance forces from the membrane itself. Membranes are a complex structure, similar to composites. They consist of a lipid bilayer, globular proteins, and a spectrin matrix, connected with a lipid bilayer by protein complexes of band 3, band 4.1, actin, and ankyrin. The property of the composite structures is their ability to acquire new mechanical properties when changing external conditions. For RBC membranes, such new properties arose at *h* > *h*_Hz_ in the spectrin matrix, since it is the main elastic structure in RBC membranes. Additional resistance forces (Figures 7 and 8) were manifested as a result of changes in the elastic properties of the spectrin matrix under the action of modifiers. So *F*(*h*) after H50 (small concentration) in 65% cases was approximated by the Hertz model, and only 35% of cases gave an increase in Young's modulus at *h* > *h*_Hz_. *F*(*h*) for hemin H80 (higher concentration) gave an increase in Young's modulus already in 94% of cases for *h* > *h*_Hz_. Both glutaraldehyde GA1% and Zn^2+^ ions also increased the membrane stiffness at depths greater than *h*_Hz_ (Figure 8, Table 2). This could be caused by the additional tension of the spectrin network and the penetration of the probe into the region of topological membrane defect (Figure 4(a)).

In 100% of cases, the empirical data curve *F*(*h*) after point *h*_Hz_ was located on the graph above the approximation curve. This means that at the depths of the RBC membrane bending larger than *h*_Hz_, the membranes became stiffer and their Young's modulus increased. Thus, to depths *h*_Hz_, modulus *E* = const and RBC membranes behaved as homogeneous elastic structures. RBCs had the maximum ability to deform. After this depth, modulus *E* increased, and for each cell according to its individual law (*b*_*n*_ and *a*_*n*_ for each cell had its own value). The membranes became stiffer. Thus, the ability of RBC to deform deeply is constant for healthy organisms and decreases with the action of modifiers and exogenous intoxications.

## 5. Conclusion

In this study, it was shown that the absolute values of Young's modulus of native RBC membranes strongly depend on the action of fixatives and factors of intoxication. The method of forming native RBC samples without fixatives was used. It was shown that under bending to depth *h*_Hz_ ≈ 0.5 *μ*m, native RBC membranes behave as homogeneous elastic structures with a constant Young's modulus. This is very important because this bending depth coincides with the typical deformation of RBC membrane in microcirculation.

The possibility of using modulus *E* as a quantitative criterion for estimating the membrane state of native cells without modifiers was discussed. At the depths of bending *h* greater than *h*_Hz_, the mechanical characteristics of the membranes are no longer described by the Hertz model. Stiffness increases according to nonlinear laws, and membranes acquire new mechanical properties. The results of the work can be used in clinical practice, in assessing the quality of stored donor blood for transfusion, in biophysical studies of RBC properties.

## Figures and Tables

**Table 1:** Young's modulus *Efor RBC membranes of healthy donors*.

Donor	1	2	3	4	5	6	7	8
*E* (kN/m^2^), sample mean *E*_m_, and standard deviation	24.9 ± 6.9	25.1 ± 3.6	27 ± 6.9	25.2 ± 4.9	25.7 ± 4.3	27.1 ± 6.3	25.8 ± 6	27.1 ± 5.6

**Table 2:** Parameters of empirical curves for Н50, Н80, GA1%, and Zn^2+^.

	No.	*b*	*a*	*E* (kPa)	*h*_Hz_ (nm)	*h*_max_ (nm)	*b*_*n*_	*a*_*n*_
H50	1	1.49	8.45*E* − 04	51.84	352	600	1.24	0.01
	2	1.50	3.42*Е* − 04	20.93	385	600	1.27	3.86*E* − 03
	3	1.51	5.48*E* − 04	33.55	600	600	1.51	5.48*E* − 04

H80	4	1.52	1.64*E* − 03	100.08	170	600	1.36	0.01
	5	1.51	5.84*E* − 04	35.78	367	600	1.07	0.02
	6	1.48	1.54*E* − 03	78.10	600	600	1.48	1.54*E* − 03

GA 1%	7	1.51	2.36*E* − 03	144.82	329	600	1.23	0.04
	8	1.51	5.65*E* − 04	34.60	463	600	1.03	0.04
	9	1.49	2.30*E* − 03	130.09	600	600	1.49	2.30*E* − 03

Zn^2+^	10	1.51	1.02*E* − 03	62.75	169	600	1.94	3.72*E* − 04
	11	1.50	1.05*E* − 03	64.38	408	600	1.39	7.66*E* − 03
	12	1.50	7.19*E* − 04	44.10	600	600	1.50	7.19*E* − 04

**Table 3:** Statistical data of the values *E* (for *h* < *h*_Hz_) and *h*_Hz_ for control and after action of agents.

	Control	H50	H80	GA1%	Zn^2+^
*E* = *E*m ± Ϭ, kPa *h* < *h*_Hz_	26.3 ± 4.8	48 ± 13	90 ± 22	119 ± 30	66 ± 19
*h*_Hz_ = *h*_Hzm_ ± Ϭ, nm	≥600 nm	421 ± 62	362 ± 79	356 ± 69	371 ± 68

## Data Availability

All data used to support the findings of this study are included within the article.
